# Crystallization and preliminary X-ray diffraction analysis of the periplasmic domain of the *Escherichia coli* aspartate receptor Tar and its complex with aspartate

**DOI:** 10.1107/S2053230X14014733

**Published:** 2014-08-27

**Authors:** Takeshi Mise, Hideyuki Matsunami, Fadel A. Samatey, Ichiro N. Maruyama

**Affiliations:** a Information Processing Biology Unit, Okinawa Institute of Science and Technology Graduate University, 1919-1 Tancha, Onna-son, Kunigami, Okinawa 904-0495, Japan; b Trans-Membrane Trafficking Unit, Okinawa Institute of Science and Technology Graduate University, 1919-1 Tancha, Onna-son, Kunigami, Okinawa 904-0495, Japan

**Keywords:** attractant, bacterial chemotaxis, cell-surface receptor, *Escherichia coli*, transmembrane signalling

## Abstract

The cell-surface receptor Tar mediates bacterial chemotaxis toward an attractant, aspartate (Asp), and away from a repellent, Ni^2+^. To understand the molecular mechanisms underlying the induction of Tar activity by its ligands, the *Escherichia coli* Tar periplasmic domain with and without bound aspartate (Asp-Tar and apo-Tar, respectively) were each crystallized in two different forms. Using ammonium sulfate as a precipitant, crystals of apo-Tar1 and Asp-Tar1 were grown and diffracted to resolutions of 2.10 and 2.40 Å, respectively. Alternatively, using sodium chloride as a precipitant, crystals of apo-Tar2 and Asp-Tar2 were grown and diffracted to resolutions of 1.95 and 1.58 Å, respectively. Crystals of apo-Tar1 and Asp-Tar1 adopted space group *P*4_1_2_1_2, while those of apo-Tar2 and Asp-Tar2 adopted space groups *P*2_1_2_1_2_1_ and *C*2, respectively.

## Abstract 2

The periplasmic domain of the *E. coli* aspartate receptor Tar was cloned, expressed, purified and crystallized with and without bound ligand. The crystals obtained diffracted to resolutions of 1.58 and 1.95 Å, respectively.

## 1. Introduction   

The aspartate receptor Tar is a transmembrane protein of 553 amino-acid residues in length consisting of a first membrane-spanning α-helix, a periplasmic domain, a second membrane-spanning α-helix and a cytoplasmic domain (Krikos *et al.*, 1983 ▶). Tar mediates bacterial chemotaxis towards attractants including aspartate (Asp) and maltose, and away from repellents such as nickel and cobalt ions (Reader *et al.*, 1979 ▶; Wang & Koshland, 1980 ▶). Transmembrane signalling by Tar requires a homodimeric structure of the receptor (Milligan & Koshland, 1988 ▶). How the attractants and repellents regulate the activity of Tar during chemotaxis is still unknown.

The first crystal structures of the Tar periplasmic domain were determined using cysteine cross-linked dimers in the absence and presence of bound aspartate in *Salmonella typhimurium* (Milburn *et al.*, 1991 ▶). Other crystal structures of the Tar periplasmic domain in the Asp-bound and unbound forms were subsequently reported (Bowie *et al.*, 1995 ▶; Chi *et al.*, 1997 ▶; Scott *et al.*, 1993 ▶; Yeh *et al.*, 1993 ▶, 1996 ▶; Yu & Koshland, 2001 ▶). Comparison of the two structures in the absence and presence of bound aspartate demonstrated a ∼1.0 Å vertical shift of the second transmembrane α-helix relative to the first (Milburn *et al.*, 1991 ▶; Chervitz & Falke, 1996 ▶; Ottemann *et al.*, 1999 ▶). It has been proposed that in transmembrane signalling, Asp binding to the periplasmic domain of Tar induces a piston-like displacement of the second transmembrane α-helix (reviewed in Falke & Hazelbauer, 2001 ▶). While the crystal structure of the apo form of the Tar periplasmic domain has been determined (Bowie *et al.*, 1995 ▶; Chi *et al.*, 1997 ▶), there has been no report of the crystal structure of the Asp-bound form of Tar from *Escherichia coli*.

Maruyama *et al.* (1995 ▶) previously proposed an alternative model in which the repellents stabilize the second transmembrane α-helix in a different rotational orientation from those of the apo and Asp-bound forms through a rotation/twist of the second transmembrane α-helix parallel to the plane of the cytoplasmic membrane. The model also predicts that apo-Tar and Asp-bound Tar have similar structures. Structural analysis of the HAMP domains, which are located immediately downstream of the second transmembrane α-helix, implies domain rotation in transmembrane signalling (Hulko *et al.*, 2006 ▶). To test the rotation/twist model, the *E. coli* Tar periplasmic domain was crystallized with and without bound aspartate (Asp-Tar and apo-Tar, respectively). Here, the expression, purification, crystallization and preliminary X-ray diffraction studies of Asp-Tar and apo-Tar are described.

## 2. Materials and methods   

### 2.1. Cloning   

A plasmid, pET-Tar, that expresses the periplasmic domain, residues 26–193 (TAR-E), of *E. coli* Tar was constructed using the expression vector pET-28a(+) (Novagen). This construct encodes an N-terminal methionine, a six-His tag and thrombin-recognition and enterokinase-recognition sites, followed by a Tar periplasmic sequence encompassing Gly26–Gln193. A DNA fragment encoding the periplasmic domain of Tar was amplified by PCR from *E. coli* DH5α genomic DNA using the forward primer 5′-ATG **GCT AGC** GAT GAC GAC GAC AAG GGC AGC CTG TTT TTT TCT TC-3′ (*Nhe*I site in bold) and the reverse primer 5′-CTC **GAA TTC** TCA TTA TTG CCA CTG GGC AAA TC-3′ (*Eco*RI site in bold). The resulting PCR product was digested with *Nhe*I and *Eco*RI, and was cloned into pET-28a(+) using a DNA ligation kit (Takara Bio, Tokyo, Japan). This construct was termed pET-Tar.

### 2.2. Expression   

An overnight culture of *E. coli* BL21 (DE3) cells (Invitrogen) harbouring pET-Tar was made by inoculating a single colony in 10 ml LB medium containing 30 µg ml^−1^ kanamycin. This overnight culture was poured into 1000 ml LB medium containing 30 µg ml^−1^ kanamycin, which was then grown at 37°C with agitation at 250 rev min^−1^ to an OD_600_ of 0.3. To this culture, isopropyl β-d-1-thiogalactopyranoside (IPTG; Nacalai Tesque, Kyoto, Japan) was added to a final concentration of 0.1 m*M* to induce the production of TAR-E. Cultivation was continued for an additional 4 h and cells were harvested by centrifugation at 6000*g* for 5 min.

### 2.3. Purification   

The harvested cell paste was resuspended in 10 ml suspension buffer (20 m*M* sodium phosphate buffer pH 7.4, 500 m*M* NaCl, 30 m*M* imidazole) with the addition of 100 µl protease-inhibitor cocktail (Nacalai Tesque) and was sonicated on ice for 40 min with an ultrasonic disruptor (Tomy Seiko, Tokyo, Japan). The resulting cell suspension was centrifuged at 6000*g* for 10 min at 4°C and resuspended in 10 ml resuspension buffer (8.0 *M* urea, 20 m*M* sodium phosphate pH 7.4, 500 m*M* NaCl) with the addition of a further 50 µl protease-inhibitor cocktail. A second sonication was carried out on ice for 1 min and the resulting suspension was pelleted by centrifugation at 20 000*g* for 15 min at 4°C. The resulting supernatant was cleared by filtration with a 0.45 µm HPLC filter (Pall Corp.).

From the cleared lysate, TAR-E was first purified by affinity chromatography with two tandemly connected 5 ml HisTrap FF columns (GE Healthcare). Before application of the sample, the columns were washed with three column volumes of washing buffer (8 *M* urea, 50 m*M* Tris–HCl pH 8.0, 200 m*M* NaCl). The cleared lysate was applied onto the HisTrap columns equilibrated with washing buffer at a flow rate of 1 ml min^−1^ for 40 min. Refolding of the bound TAR-E was performed by applying a linear urea gradient from 8.0 to 0.0 *M* in buffer consisting of 50 m*M* Tris–HCl pH 8.0, 200 m*M* NaCl at a flow rate of 1.0 ml min^−1^ for 60 min. Elution of refolded proteins was performed by applying a linear imidazole gradient ranging from 0 to 500 m*M* in buffer consisting of 50 m*M* Tris–HCl pH 8.0, 200 m*M* NaCl at a flow rate of 1 ml min^−1^ for 60 min. Eluted TAR-E was further purified by gel-filtration chromatography with a HiLoad column (26/600 Superdex 75 pg; GE Healthcare) equilibrated with 1.0 m*M* EDTA, 10 m*M* Tris–HCl pH 8.0, 100 m*M* NaCl by FPLC at a flow rate of 1.0 ml min^−1^. Fractions containing TAR-E, ∼30 ml, were dialyzed against buffer consisting of 0.6 *M* NaCl, 10 m*M* HEPES–NaOH pH 7.5 and were concentrated by ultrafiltration using Vivaspin 6 (Vivaproducts, Massachusetts, USA). Protein purity was assessed by SDS–PAGE (14%), followed by staining with Coomassie Brilliant Blue. Concentrated TAR-E at 11.6–15.9 mg ml^−1^ was immediately used for crystallization without freezing. During cleavage with thrombin or enterokinase in order to remove the tag, His-tagged TAR-E precipitated for unknown reason(s). Therefore, TAR-E protein was directly used for crystallization without protease digestion.

### 2.4. Crystallization using ammonium sulfate as a precipitant   

**Figure 1:**
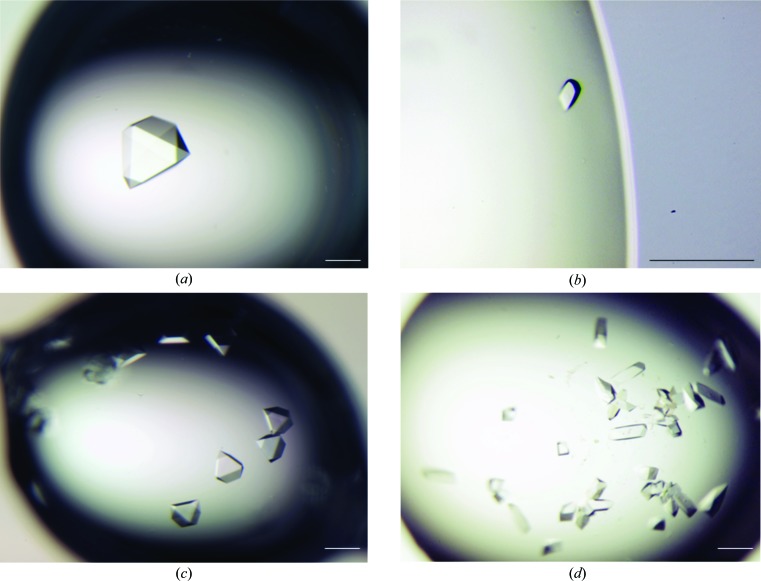
Crystals of apo-Tar1 (*a*), apo-Tar2 (*b*), Asp-Tar1 (*c*) and Asp-Tar2 (*d*). The scale bar is 200 µm in length.

All crystallization experiments were performed in 24-well VDX plates with sealant (Hampton Research, California, USA) against a 1.0 ml reservoir. Previously reported conditions for crystallization (Bowie *et al.*, 1995 ▶) were employed with slight modification. Crystals of apo-Tar1 were grown by mixing 1.5 µl purified TAR-E protein solution (0.6 *M* NaCl, 10 m*M* HEPES–NaOH pH 7.5, 15.9 mg ml^−1^ TAR-E) with 1.5 µl crystallization solution [0.4 *M* ammonium sulfate, 35 m*M* ammonium formate, 15 m*M* formic acid, 1.25%(*v*/*v*) glycerol pH 3.9] on a siliconized glass cover slide at 10°C. Hanging drops consisting of 1.5 µl each of the protein and crystallization solutions were equilibrated against a 1.0 ml reservoir of crystallization solution. A crystal of apo-Tar1 was sequentially soaked in crystallization solution supplemented with 10, 20 or 30%(*v*/*v*) glycerol and was flash-cooled in liquid nitrogen (Fig. 1 ▶ *a*).

Prior to crystallization of Asp-Tar1, 44 µl of an aspartate solution (0.6 *M* NaCl, 10 m*M* HEPES–NaOH pH 7.5, 45 m*M* aspartate) was mixed with 156 µl purified TAR-E protein solution (0.6 *M* NaCl, 10 m*M* HEPES–NaOH pH 7.5, 11.6 mg ml^−1^ TAR-E). Asp-Tar1 crystals were grown at 10°C by mixing 1.5 µl purified TAR-E solution (0.6 *M* NaCl, 10 m*M* HEPES–NaOH pH 7.5, 9.9 m*M* aspartate, 9.1 mg ml^−1^ TAR-E) with 1.5 µl crystallization solution (0.8 *M* ammonium sulfate, 20 m*M* ammonium formate, 30 m*M* formic acid pH 3.4) on a siliconized glass cover slide with streak-seeding with previously obtained Asp-Tar1 crystals. Hanging drops consisting of 1.5 µl each of the protein and crystallization solutions were equilibrated against a 1.0 ml reservoir of crystallization solution. A crystal of Asp-Tar1 was sequentially soaked in crystallization solution supplemented with 20, 30 or 35%(*v*/*v*) glycerol and was flash-cooled in liquid nitrogen (Fig. 1 ▶ *c*).

### 2.5. Crystallization using NaCl as a precipitant   

For crystallization of apo-Tar2 and Asp-Tar2, NaCl was also used as a protein precipitant. Crystallization conditions with modifications have previously been reported (Chi *et al.*, 1997 ▶). Crystals of apo-Tar2 were grown at 10°C by mixing 1.5 µl purified TAR-E solution (0.6 *M* NaCl, 10 m*M* HEPES–NaOH pH 7.5, 13.7 mg ml^−1^ TAR-E) with 1.5 µl crystallization solution (4.3 *M* NaCl, 100 m*M* Tris–HCl pH 8.0) on a siliconized glass cover slide with streak-seeding with crystals of apo-Tar1. Hanging drops consisting of 1.5 µl each of the protein and crystallization solutions were equilibrated against a 1.0 ml reservoir of crystallization solution. A crystal of apo-Tar2 was soaked in crystallization solution supplemented with 33%(*v*/*v*) glycerol and was flash-cooled in liquid nitrogen (Fig. 1 ▶ *b*).

Crystals of Asp-Tar2 were grown at 10°C by mixing 1.5 µl purified TAR-E solution (0.6 *M* NaCl, 10 m*M* HEPES–NaOH pH 7.5, 13.5 mg ml^−1^ TAR-E) with a 1.5 µl reservoir of crystallization solution (3.5 *M* NaCl, 100 m*M* Tris–HCl pH 8.0, 25 m*M* aspartate) on a siliconized glass cover slide with streak-seeding with crystals of apo-Tar1. Hanging drops consisting of 1.5 µl each of the protein and crystallization solutions were equilibrated against a 1.0 ml reservoir of crystallization solution. A crystal of Asp-Tar2 was sequentially soaked in a crystallization solution supplemented with 25 or 33%(*v*/*v*) glycerol and was flash-cooled in liquid nitrogen (Fig. 1 ▶ *d*).

### 2.6. X-ray diffraction data collection   

X-ray diffraction data were collected under cryogenic conditions by flash-cooling with liquid nitrogen after stepwise transfers of all crystals into the crystallization solution supplemented with 10–35%(*v*/*v*) glycerol. Diffraction data for apo-Tar1 and Asp-Tar1 were collected using an ADSC Q270 detector on the AR-NE3A beamline and an ADSC Q315 detector on the BL5A beamline at the Photon Factory, Tsukuba, Japan, respectively. Diffraction data for apo-Tar2 and Asp-Tar2 were collected using a MAR 300HE charge-coupled device (CCD) detector on the BL44XU beamline and an ADSC Quantum315 CCD detector on the BL38B1 beamline at SPring-8, Harima, Japan, respectively. Diffraction images were processed with *iMosflm* and scaled using *SCALA* in the *CCP*4 program suite (Battye *et al.*, 2011 ▶; Winn *et al.*, 2011 ▶). The crystal data statistics are listed in Table 1 ▶.

## 3. Results and discussion   

**Figure 2:**
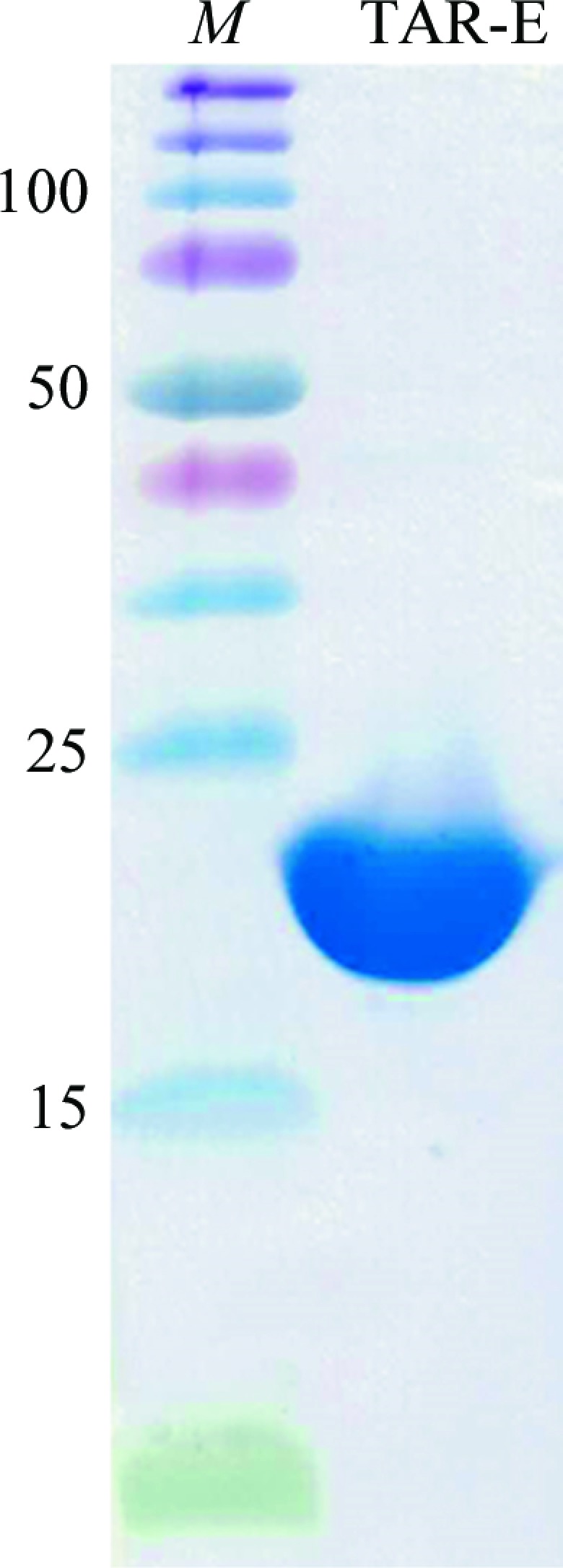
SDS–PAGE analysis of purified TAR-E. Lane *M* contains molecular-mass standards (labelled in kDa).

The TAR-E expression plasmid encodes the entire periplasmic domain of Tar from Gly26 to Gln193, which is the longest of the Tar periplasmic domains that have been crystallized to date (Bowie *et al.*, 1995 ▶; Chi *et al.*, 1997 ▶; Jancarik *et al.*, 1991 ▶; Milburn *et al.*, 1991 ▶; Scott *et al.*, 1993 ▶; Yeh *et al.*, 1993 ▶, 1996 ▶; Yu & Koshland, 2001 ▶). TAR-E was purified to give a single band on SDS–PAGE (Fig. 2 ▶). The molecular mass of denatured TAR-E was estimated to be 22 kDa, which is comparable to the calculated mass (22.3 kDa).

**Figure 3:**
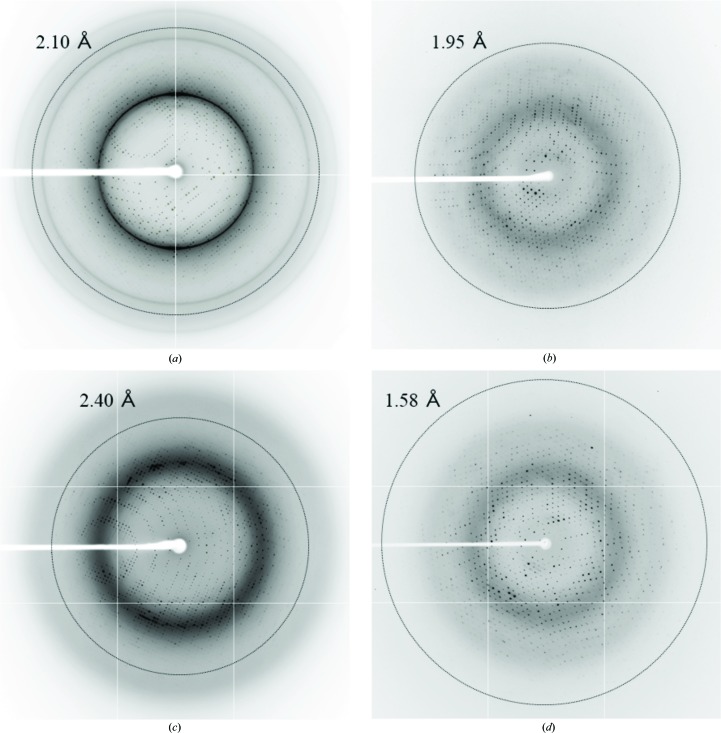
X-ray diffraction images from crystals of apo-Tar1 (*a*), apo-Tar2 (*b*), Asp-Tar1 (*c*) and Asp-Tar2 (*d*). Resolution circles are also shown with the corresponding values.

Asp-Tar1 and apo-Tar1 were crystallized in the presence and absence of Asp, respectively (Fig. 1 ▶). Based on previously reported methods (Bowie *et al.*, 1995 ▶), the crystallization conditions for apo-Tar1 were optimized by varying the ammonium sulfate concentration in the range 0.4–1.1 *M*, by varying the buffer pH from pH 3.6 to 3.9 and by varying the incubation temperature from 10 to 25°C. Crystal nuclei appeared in 7 d in a droplet after crystallization commenced. The best crystal diffracted to 2.10 Å resolution and adopted space group *P*4_1_2_1_2 (Fig. 3 ▶ *a*, Table 1 ▶). For crystallization of Asp-Tar1 (Fig. 1 ▶ *c*), the conditions were optimized by varying the ammonium sulfate concentration in the range 0.4–0.8 *M* and the buffer pH from pH 3.4 to 3.9. Crystal nuclei appeared within 2 d in a droplet. The crystal of Asp-Tar1 diffracted to 2.40 Å resolution (Fig. 3 ▶ *c*) and adopted space group *P*4_1_2_1_2 (Table 1 ▶).

Bowie *et al.* (1995 ▶) reported that sulfate ions occupied aspartate-binding sites in the crystals of apo-Tar when ammonium sulfate was used as a precipitant. To overcome this problem, NaCl was used as a precipitant instead of ammonium sulfate (Figs. 1 ▶ *b* and 1 ▶ *d*). It took a few months to obtain crystals of apo-Tar2. On the other hand, many crystal nuclei of Asp-Tar2 appeared within 7 d in a droplet after the crystallization commenced. Crystals of apo-Tar2 and Asp-Tar2 diffracted to 1.95 and 1.58 Å resolution, respectively (Figs. 3 ▶ *b* and 3 ▶ *d*), and adopted space groups *P*2_1_2_1_2_1_ and *C*2, respectively (Table 1 ▶). Structure determinations of all four types of crystals (apo-Tar1, apo-Tar2, Asp-Tar1 and Asp-Tar2) are currently in progress, and we have confirmed that aspartate molecules are bound in the crystals of Asp-Tar1 and Asp-Tar2 (data not shown). Furthermore, crystallization of the periplasmic domain with bound Ni^2+^ is advancing.

## Figures and Tables

**Table 1:** Data-collection and processing statistics Values in parentheses are for the outermost resolution shell.

Data collection	apo-Tar1	apo-Tar2	Asp-Tar1	Asp-Tar2
X-ray source	AR-NE3A PF	BL44XU SPring-8	BL5A PF	BL38B1 SPring-8
Detector	ADSC Q270	MAR300HE	ADSC Q315	ADSC Q315
Wavelength (Å)	1.000	0.900	1.000	1.000
Beam size (µm)	200 (diameter)	30.0 (diameter)	200 (diameter)	180.0 (vertical) × 88.0 (horizontal)
Crystal-to-detector distance (mm)	213.6	210.0	249.2	200.0
Oscillation angle (°)	1	1	1	1
Oscillation range (°)	180	180	180	180
Exposure time (s)	2.0	1.1	2.0	2.2
Space group	*P*4_1_2_1_2	*P*2_1_2_1_2_1_	*P*4_1_2_1_2	*C*2
Unit-cell parameters				
*a* (Å)	85.07	60.65	84.49	81.11
*b* (Å)	85.07	73.60	84.4	59.76
*c* (Å)	106.69	79.13	105.47	79.16
α (°)	90.00	90.00	90.00	90.00
β (°)	90.00	90.00	90.00	94.84
γ (°)	90.00	90.00	90.00	90.00
Molecules in asymmetric unit	2	2	4	4
*V*_M_ (Å^3^ Da^−1^)	2.17	1.98	2.11	2.15
Solvent content (%)	43.3	38.0	41.9	42.8
Mosaicity (°)	0.48	0.63	0.94	0.52
Resolution range (Å)	2.10–32.00 (2.10–2.21)	1.95–36.80 (1.95–2.06)	2.40–39.54 (2.40–2.53)	1.58–31.26 (1.58–1.67)
Measured reflections	291572 (46683)	191802 (27700)	194541 (27924)	173532 (26746)
Unique reflections	21720 (3349)	26480 (3792)	15497 (2197)	51023 (7475)
Completeness (%)	92.5 (100.0)	99.9 (100.0)	99.7 (100.0)	98.7 (99.4)
*R*_merge_ † (%)?	0.061 (0.144)	0.080 (0.293)	0.062 (0.255)	0.145 (0.278)
Multiplicity	13.4 (13.9)	7.2(7.3)	12.6 (12.7)	3.4 (3.6)
Mean *I*/σ(*I*)	32.1 (16.0)	16.7 (7.1)	28.6 (10.2)	5.8 (3.6)

† *R*_merge_ = 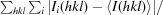 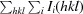, where *I_i_*(*hkl*) is the observed intensity of an individual reflection and 〈*I*(*hkl*)〉 is the mean intensity of that reflection.
